# A novel low-cost uterine balloon tamponade kit to tackle maternal mortality in low-resource settings

**DOI:** 10.1038/s41598-024-60064-z

**Published:** 2024-05-01

**Authors:** Sara Candidori, Kasra Osouli, Serena Graziosi, Alberto Antonio Zanini, Maria Laura Costantino, Francesco De Gaetano

**Affiliations:** 1 Department of Mechanical Engineering, Politecnico Di Milano, Via La Masa 1, 20156 Milan, Italy; 2 Department of Chemistry, Materials and Chemical Engineering “G. Natta”, Politecnico Di Milano, Piazza Leonardo da Vinci 31, 20133 Milan, Italy; 3 Obstetrician Gynaecologist, Freelance Professional, Milan, Italy

**Keywords:** Biomedical engineering, Health care, Mechanical engineering

## Abstract

The 3.1 target of the Sustainable Development Goals of the United Nations aims to reduce the global maternal mortality ratio to less than 70 maternal deaths per 100,000 live births by 2030. The last updates on this target show a significant stagnation in the data, thus reducing the chance of meeting it. What makes this negative result even more serious is that these maternal deaths could be avoided through prevention and the wider use of pharmacological strategies and devices to stop postpartum haemorrhage (PPH). PPH is the leading obstetric cause of maternal mortality in low- and middle-income countries (LMICs). Despite low-cost devices based on the uterine balloon tamponade (UBT) technique are already available, they are not safe enough to guarantee the complete stop of the bleeding. When effective, they are too expensive, especially for LMICs. To address this issue, this study presents the design, mechanical characterisation and technology assessment performed to validate a novel low-cost UBT kit, particularly a novel component, i.e., the connector, which guarantees the kit’s effectiveness and represents the main novelty. Results proved the device’s effectiveness in stopping PPH in a simulated scenario. Moreover, economic and manufacturing evaluations demonstrated its potential to be adopted in LMICs.

## Introduction

Maternal mortality—the death of a woman while pregnant or within 42 days of termination of pregnancy^1^– is a major challenge to health systems worldwide^2^. Global initiatives to tackle maternal mortality started almost half a century ago with the Safe Motherhood Initiative^3^. The focus on this issue was strengthened when reducing maternal mortality became one of the eight Millennium Development Goals (MDGs, Goal 5)^4^. The target was to reduce the maternal mortality ratio (MMR) by 75% between 1990 and 2015. Despite a significant reduction in maternal deaths—from an estimated 523,000 deaths in 1990 to 289,000 in 2013, the ratio dropped by less than half of what was needed to achieve the MDG target (from 380 to 210 maternal deaths per 100,000 live births)^5^. After MDGs failed, the Sustainable Development Goals (SDGs) agenda, approved in 2015, renewed the focus on maternal health^6^. The first target of Goal 3 was again the reduction of the global MMR to the more ambitious target of less than 70 by 2030, with no country with an MMR of more than 140. However, the latest published trends estimated that 287,000 women and adolescent girls died because of pregnancy and childbirth-related complications in 2020 globally, equivalent to approximately one death every two minutes^1^. Comparing these data with those reported for the MDG period, it is evident that a strong stagnation happened during the first years of the SDG era.

Although the global trends described above are already dramatic, what makes them even more tragic is that almost 95% of all maternal deaths occurred in low- and middle-income countries (LMICs) in 2020, and most of them could have been prevented^1^. Besides, while most cases of maternal death in high-income countries (HICs) are unfortunate, isolated medical incidents, the same cannot be said for LMICs. Sub-Saharan Africa alone accounted for approximately 70% of global maternal deaths in 2020, with a regional MMR estimated at 545 maternal deaths per 100,000 live births. This value is 136 times higher than the MMR in Australia and New Zealand (equal to 4)^1^.

The clinical knowledge, practice, and technology for preventing maternal deaths have existed for decades, but unfortunately, too many women still lack access to life-saving solutions^1^. These solutions are indeed often not available, not accessible or not implemented, especially in low-resource settings (LRSs). The last report from the WHO also pointed out that a substantial variation in the burden of maternal mortality across regions was apparent according to income groups^1^, documenting the influence of social and economic determinants.

Maternal deaths can be due to multiple causes, ranging from proximate (i.e., medical) direct obstetric causes (e.g., pregnancy complications) or indirect causes (e.g., infectious and non-communicable diseases) to distal causes, such as health system failures^1^. Among direct obstetric causes, haemorrhage, especially postpartum haemorrhage (PPH), is the leading cause, representing more than one-quarter of global maternal deaths^7–9^. PPH, which is commonly defined as a blood loss of at least 500 ml within 24 h after birth^10^, can be prevented by adopting active management of the last stage of labour based on the use of uterotonic drugs^10,11^. If prevention is not effective or not applied, PPH can be managed in HICs with first-line interventions, such as uterotonic drugs, tranexamic acid, and intravenous fluids^10,12^. In case of unresponsive bleeding, second-line treatments such as uterine balloon tamponade (UBT) devices, uterine arterial embolisation and surgery can be employed^10^. However, access to these critical interventions is often lacking in LRSs, contributing to the high morbidity and mortality rates attributed to PPH^13,14^. Moreover, as documented in^15^, obstetric education and training quality also play a valuable role in reducing PPH mortality.

**Figure 1:**
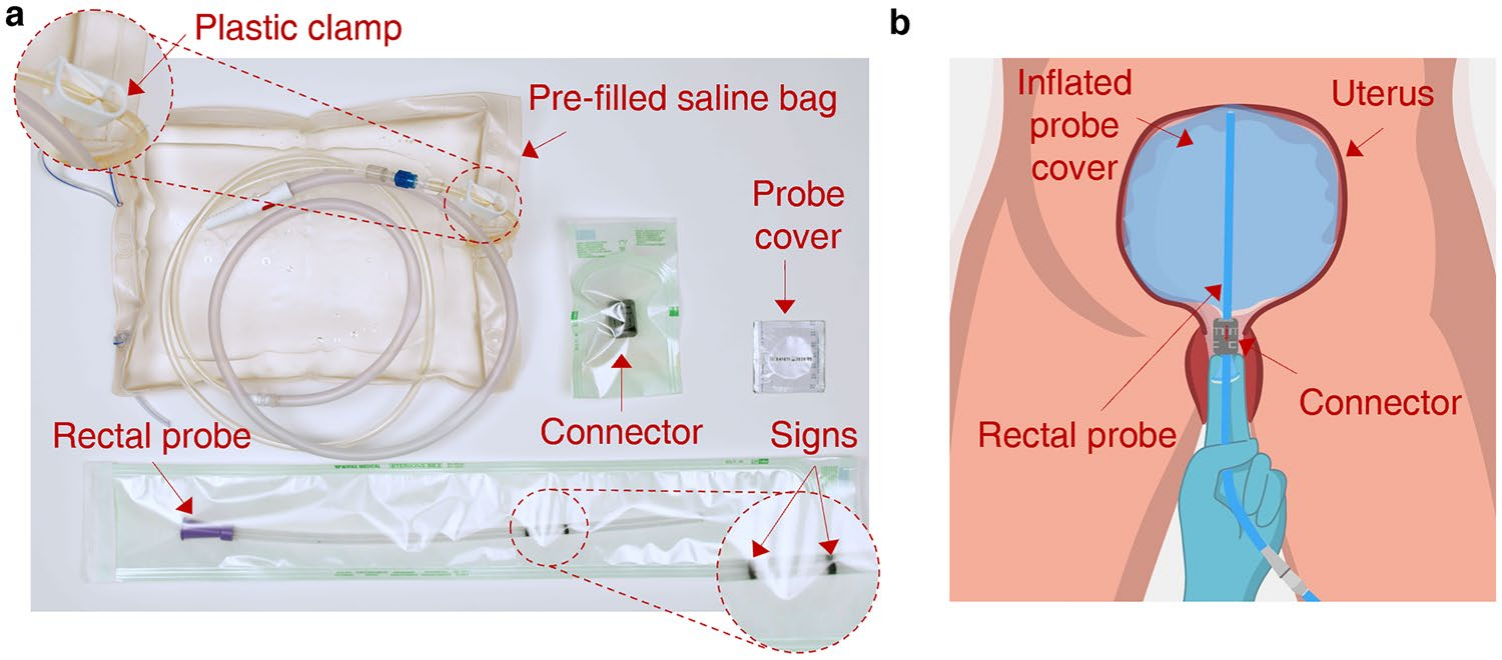
Components of the BAMBI kit and its mechanism of use. (**a**) The BAMBI kit is composed of a pre-filled saline bag equipped with a plastic clamp, a probe cover (condom), a connector, and a rectal probe. Image adapted from^22^ and used under CC BY 4.0. (**b**) Mechanism of use: the assembled device is inserted into the uterus through the vaginal canal and inflated to stop the bleeding.

To promote a more widespread diffusion of second-line treatments, especially in LRSs, this paper details the design and testing phases of a novel element of a low-cost UBT kit called BAMBI (Balloon Against Maternal BleedIng). The BAMBI kit comprises (Fig. 1a): an ultrasound probe cover, a rectal probe, a connector to secure the probe cover to the rectal probe, and a urinary drain bag pre-filled with saline solution or sterile water and equipped with a plastic clamp. Except for the connector, which is the focus of this study and has been patented^16^, all the other elements are medical devices commercially available at low cost; they are daily used in clinical practice (also in field hospitals in LMICs), have standardised sizes and are sold sterile. To be precise, the BAMBI kit is based on the condom balloon tamponade (CBT) technique. Due to the unavailability, in LRSs, of commercial pre-assembled UBT devices (such as the golden standard Bakri® Postpartum Ballon^17^ or the cheaper Ellavi^18,19^), low-cost adaptations based on the use of condoms have emerged in those areas of the world. In the CBT technique, a condom or probe cover is tied to a catheter through sutures or strings, inserted into the uterus, and filled with saline solution or sterile water to stop the bleeding. Although potentially life-saving, CBT has relevant limitations, such as fluid leakages and the need for specific training and manual skills to perform knots. To address these limitations, BAMBI has been conceived with a purposely designed connector, which allows the condom to be assembled to the catheter in a more reliable and easier-to-assemble way than sutures (Fig. 1b). In addition, the principles of frugal engineering have been implemented for its design (i.e., “*achieving more with fewer resources*”^20,21^) because they align with the study's purpose. Details about the connector design, experimental validation, production costs, and training modalities are provided in this paper. Besides, a discussion of how further improvements have been applied to the connector design based on the feedback collected by medical and non-medical users involved in usability tests (performed by the authors in a previous study and already described in^22^) is also provided.

## Methods

The development of the connector of the BAMBI kit (Fig. 1a) started with a detailed analysis of the main design requirements to be considered. Then, prototypes were fabricated and tested. The experimental tests focused on evaluating the connection strength through mechanical tensile testing and the measurement of the intraluminal pressure. Both tests were relevant to evaluating the connector's (and the kit's) performance and collecting design feedback to improve its design. It should be underlined that no standard validation procedures are available for UBT devices. The only publicly available information can be found in^23^ and refers to the test performed on the ESM™ device. This low-cost CBT kit contains everything needed to perform the procedure, with O-rings that substitute surgical sutures^24–27^. That study^23^ was therefore taken as a reference for evaluating the connection strength and measuring the intraluminal pressure.

### Design and prototyping

Once the main design requirements for the connector and the whole BAMBI kit were settled, the software SOLIDWORKS® (by Dassault Systèmes) was used to create the digital models of the developed concepts. These concepts were prototyped for preliminary functional evaluations using a PLA filament and the Kenstrapper Verve^28^ 3D printer (fused filament fabrication (FFF)) equipped with a 0.4 mm extruder. The Form 3B^29^ 3D printer (stereolithography (SLA)) and the Formlabs Grey Resin (Formlabs Inc.) were instead used to manufacture the prototypes used for the validation tests.

Commercial and standard products were selected for the other elements of the BAMBI kit shown in Fig. 1a (i.e., the urinary drain bag, rectal probe/catheter, probe cover, and plastic clamp). Unlike standard CBT solutions involving a catheter (typically a urinary Foley catheter)^30^, a 22 CH rectal probe from ConvaTec® was used as an introducer. We selected rectal probes (that are elongated semi-flexible tubes used for rectal administration of medications and fluid drainage) because they are suitable both in terms of material and size, have an attachment compatible with urine collection bags, are less flexible compared to catheters (this feature facilitates their insertion into the uterus), have a soft distal tip (which reduces the risk of uterine wall damage), and are cheaper compared to catheters. The probe cover is a condom-like latex cover regularly used as a physical barrier to prevent cross-contamination during ultrasound exams. Urinary drain bags are collection bags attached to a catheter (a flexible tube inserted into the bladder) to collect urine.

### Evaluation of connection strength

When a UBT device is inserted and inflated into the uterus (Fig. 1b), it is subjected to forces that can compromise its integrity, causing the probe cover to detach from the rectal probe, leading to leakage and depressurisation of the balloon and putting the patient’s life in danger. Tensile tests were performed according to the protocol proposed in^23^ to evaluate the connection strength for different designs and analyse the influence of design variables on this strength for the most promising concept.

**Figure 2:**
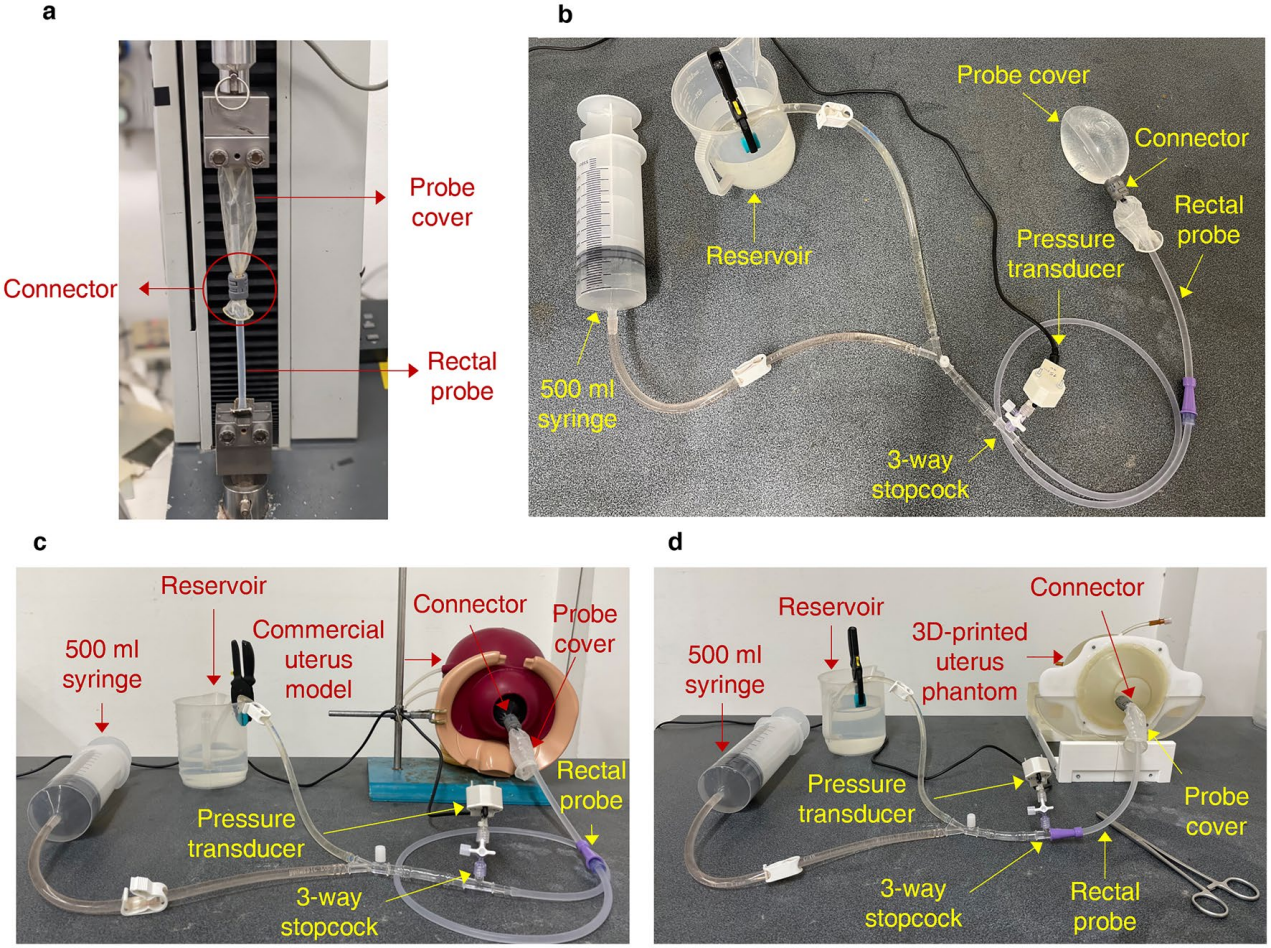
Mechanical characterisation of the BAMBI device: the multiple experimental setups used. (**a**) Evaluation of the connection strength through tensile testing. (**b)** Intraluminal pressure measurement in open air condition. (**c-d**) Intraluminal pressure measurement in confined conditions: a commercial uterus model (**c**) and an ad hoc 3D-printed uterus phantom (**d**)^31^. The setups in **b-d** comprise a 500 ml syringe, a water reservoir, a pressure transducer, and a 3-way stopcock for tubing connections.

The MTS Synergie 200H (MTS Systems Corporation) electromechanical test system, equipped with a 100N load cell, was used (Fig. 2a). The load was measured by a load cell mounted on a mobile crossbar. The load cell was connected to the distal end of the probe cover. The proximal end of the rectal probe was connected to the bottom clamp. Covering the extremities with an elastic membrane (natural rubber sheet, thickness 0.5 mm) improved the grip and protected the probe cover and the rectal probe from damage and rupture during the test.

All tests were conducted at room temperature, with the crosshead moving at 100 mm/min until failure and at a 60 Hz acquisition rate for force and displacement values. Failure was defined as the probe cover started slipping on the probe, as indicated by a sudden decrease in the measured force. If no slippage occurred, tests were terminated at the maximum elongation of 500 mm. Data were post-processed in Matlab®. Each test was repeated three times. The force's mean and standard deviation values were calculated for each displacement. The mean load–displacement curves and error bars (± standard deviation) were plotted at selected displacement values.

### Intraluminal pressure measurement

The intraluminal pressure (ILP) is the pressure inside the balloon, evaluated as a function of the filling volume and the external conditions, i.e., the environment in which the balloon inflates. As discussed in a previous paper^31^, this pressure also depends on the stiffness of the material the balloon is made of. Reference values for UBT solutions based on the use of condoms/probe covers can be found in^23^ and refer again to the mechanical characterisation of the ESM.

To evaluate the ILP developed in the balloon at different filling stages, the setup shown in Fig. 2b–d was used for the open-air (Fig. 2b) and confined conditions (Fig. 2c,d). In all cases, the rectal probe of the BAMBI kit was connected through a hydraulic circuit to a 500 ml syringe and a water reservoir. A 3-way stopcock allowed the inflation of the balloon by injecting water from the reservoir at 50 ml steps through the syringe. A pressure transducer (42PC15D pressure sensors, Honeywell Inc.®) measured the ILP at each volume step. The measured ILP values were collected through the NI USB-6218 DAQ board (National Instruments™) using LabVIEW™ software. All tests were carried out at room temperature.

For the confined conditions, the commercial simulator Prompt Flex PPH Module LIM-80101 (Limbs and Things Inc.™) (Fig. 2c) and a 3D-printed uterus phantom (Fig. 2d and Supplementary Fig. S1) were used. The procedure was repeated until 1500 ml of water was injected into the balloon. The maximum water volume was reduced to 700 ml in the confined tests with the commercial simulator. This value corresponds to the volume of the internal cavity of the commercial uterus. Details about the 3D-printed phantom can be found in a previous work^31^. Compared to the commercial simulator, the 3D-printed uterus is bigger and has an internal cavity of approximately 2500 ml. It comprises a single uterus body that can be assembled with three interchangeable cervices, characterised by different dilations (see^31^ and Supplementary Fig. S1a). The “medium size” cervix was used for the tests, corresponding to a dilation of 50 mm^31^. The phantom is equipped with a reclinable support to mimic three physiological inclinations of the uterus during childbirth (see^31^ and Supplementary Fig. S1b). Support clamps were used to simulate this inclination for the commercial model. The most critical one, for using a UBT device, was selected in both cases. For the 3D-printed phantom model, it corresponds to an inclination of 100° (see^31^ and Supplementary Fig. S1b).

The ILP was also measured during a 4-h test in confined conditions (commercial uterus model) to verify if the plateau pressure value was maintained over time, similar to what was found for the ESM device^23^. The balloon was inflated to 700 ml of water and left inside the uterus model for 4 h, with the ILP measured every hour (Fig. 2c).

## Results

### Design requirements definition and preliminary prototypes

The design requirements, briefly described by the authors in a previous work^32^, are further detailed here. They were first defined for the connector and then for the whole kit.

The design requirements for the connector include:

- *Hydraulic sealing*: the connector must guarantee the absence of leakages for the whole duration of its action (i.e., at least 3 h, as tested in^23^);
- *Connection strength*: the connection must not be disassemblable during its working time (tensile force at failure > 15 N, reference value from^23^);
- *Biocompatibility and sterilisation of the materials*: since the connector has to be inserted through the vaginal canal into the uterus (Fig. 1b), it is considered an invasive tool. Hence, it must be manufactured with medical-care materials (e.g., polyethylene, PE) and must be sterilisable;
- *Minimum size and anatomical compatibility*: during the insertion of the device, the connector is the part with the largest radius, which should be compatible with the anatomical dimension of the cervicovaginal funnel even in the absence of the dilation typical of the labour; a maximum external diameter of 20 mm is set, and sharp edges should be avoided to prevent internal wounds (${\varnothing }_{max}\le 20 mm$);
- *Minimum cost*: a target *production* cost of €1/piece has been set. This value has been considered suitable for producing a low-cost kit.

The design requirements for the whole BAMBI kit include:

- *Efficacy and effectiveness*: the device must apply proper pressure against the internal uterine walls. A target intraluminal pressure of 12.5 mmHg in open air (comparable to already approved devices^23^) has been set. This value has to be verified through in vitro experiments. More in general, the target of the device is to stop bleeding in real PPH-related emergencies; on-field clinical trials will be necessary;
- *Rapidity to assembly*: the device must be assembled as fast as possible due to the emergency conditions; this requirement should be fulfilled through an appropriate design of the kit and training of the operators. Reference assembly times are not available for other CBT solutions; a target of less than 2 min is defined, considering that, given a copious blood loss of 500 ml/min, it corresponds to a haemorrhage of 1,000 ml (target assembly time < 120 s);
- *Easy to use*: the device should be assembled and used even by not qualified operators; it is also preferable that a single operator performs the whole procedure;
- *Safety, reliability and quality*: the device must perform its intended function without failure, errors, or breakdowns over a specified period and under normal operating conditions;
- *Minimum cost*: a maximum market cost of €5/kit has been set. This target has been determined to have a market cost lower than the Ellavi device (US$7.50^33^) and comparable to the ESM (less than US$5^24^).

Transversally to these requirements, the BAMBI kit should be affordable, accessible, and acceptable. These requirements are intimately related to the specific clinical condition and use context (treating PPH in LRSs). They include economic considerations but also social and ethical ones. A frugal engineering approach has been pursued by adopting a few broadly available cheap components in the kit (probe cover, rectal probe, and urinary bag, Fig. 1a), which positively influences the accessibility and affordability of the BAMBI device.

In the initial phase of the design process, various solutions for the connector were developed, taking the identified requirements as a reference. The most promising ones were included in the patent application^16^, ranging from hinge- or membrane-based solutions to auxetic structures. Among them, three were selected for an initial validation phase: the “*interlocking connector*”, the “*hinge connector*”, and the “*rubber band connector*”. More details about each connector's components, assembly and use procedure, and preliminary tests performed to choose the most promising one are provided in the Supplementary Note—Preliminary concepts. Based on these evaluations, the rubber band-based design was deemed the most appropriate for the specific application. It was thus studied extensively in the second phase of the design process, and it is described in detail in the next section.

### Detailed design of the selected connector and mechanical characterisation

**Figure 3:**
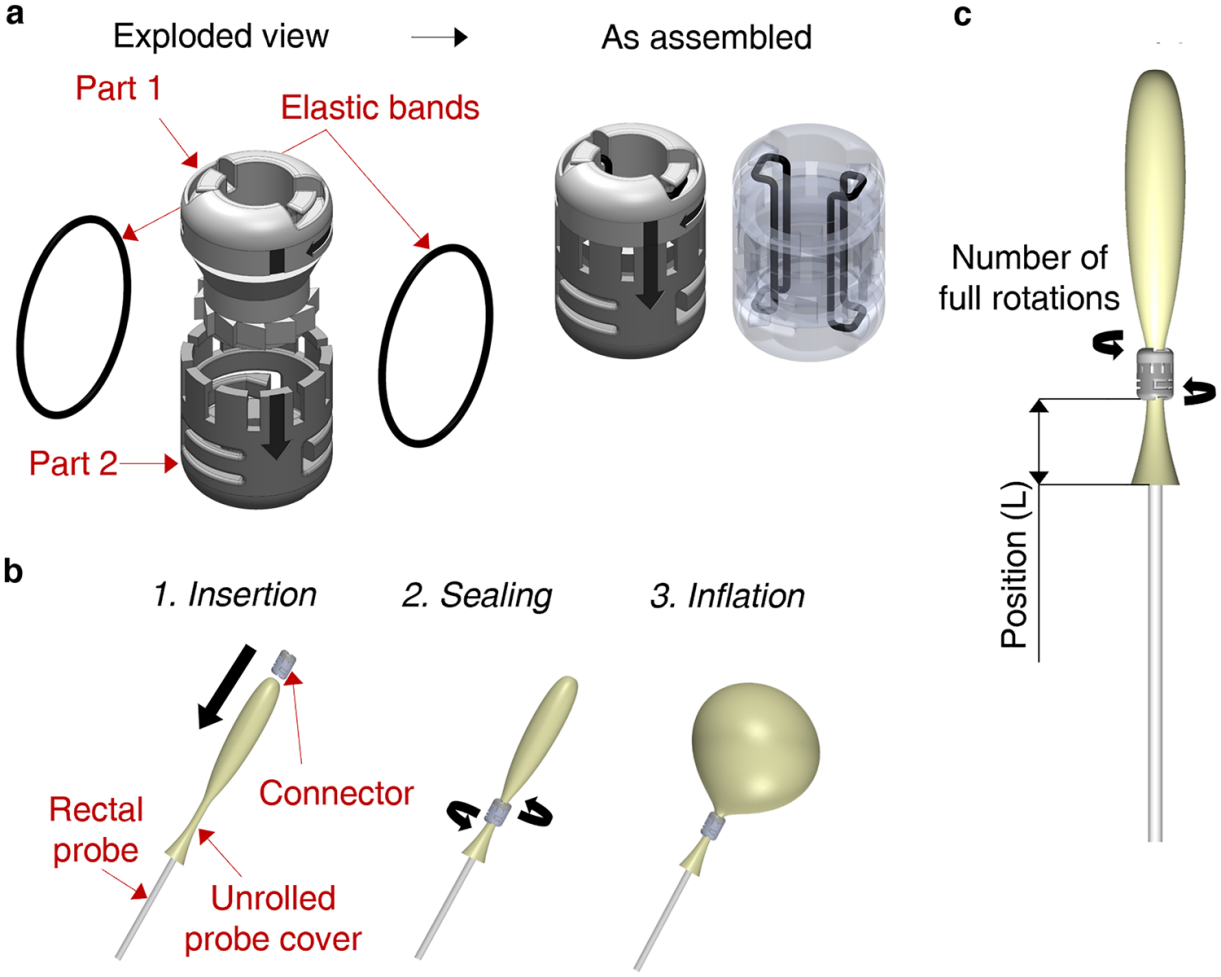
The BAMBI connector. (**a**) Connector exploded view (left) and as assembled (right). It consists of two parts and two elastic bands, and it is already pre-assembled. (**b**) Assembly procedure of the connector to the rectal probe and the probe cover: 1. Insertion of the connector over the rectal probe and the unrolled probe cover; 2. Rotation of part 1 with respect to part 2 to wind the elastic bands around the probe cover and the rectal probe; 3. Inflation of the probe cover. (**c**) How the “L” parameter is measured.

The rubber band-based connector (Fig. 3a) comprises two components (called part 1 and part 2), one of which rotates within the other. Two elastic bands from each side are attached to the two parts. The opposite rotation of the parts wraps the elastics tightly around the probe cover and the rectal probe. A rotary ratchet mechanism allowing only one-way rotation of the parts was conceived to prevent the elastic bands from unwinding while in tension. The connector is already assembled, and the user has only to assemble the BAMBI kit (Fig. 1a), as shown in Fig. 3b. After having unrolled the probe cover over the distal tip of the rectal probe (until its extremity touches the rectal probe), the user must insert the connector and perform few rotations to fix it before inflation. As explained later, the exact number of rotations has been derived experimentally. The connector has an inner diameter of 10 mm and an outer diameter of 20 mm with no sharp edges (see Supplementary Fig. S2); hence, it satisfies the *minimum size and anatomical compatibility* requirements. This dimensional check was performed with the contribution of a gynaecologist on the research team.

However, to complete this concept's design, some aspects concerning the connection strength and position along the rectal probe had to be further investigated. To this aim, two design variables were considered (Fig. 3c):

1. the number of full rotations (360 degrees) of part 1 of the connector with respect to part 2 (1, 2, or 3 full rotations);
2. the position of the connector from the proximal end of the probe cover, “L” (Fig. 3c): 50, 75, and 100 mm.

Figure 3c shows how the “L” parameter is measured after the insertion of the probe cover following the instructions shown in Fig. 3b. The target was to decide where to put the two black signs visible in the zoomed view in Fig. 1a, which are fundamental for guiding the user in correctly positioning the connector.

**Figure 4:**
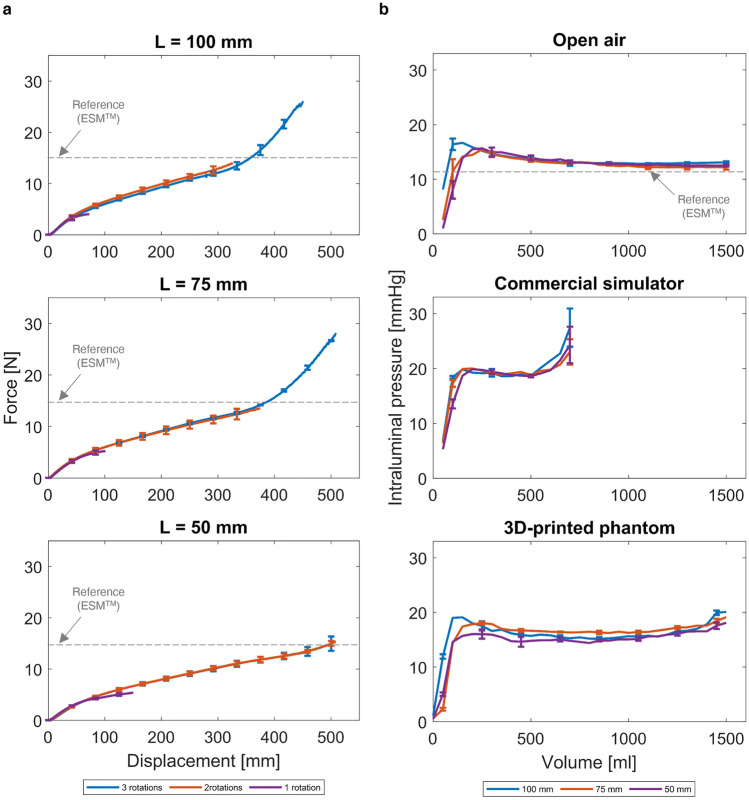
Mechanical characterisation of the BAMBI device and comparison with reference values from^23^ related to the ESM™ device. (**a**) Evaluation of the connection strength through tensile testing. Force–displacement curves of the assembled device as a function of the number of full rotations and connector position. Three samples were tested for each configuration. Mean curves and error bars (± standard deviation) are reported at selected displacement values. (**b**) Intraluminal pressure–volume curves in the open air and confined conditions (commercial simulator and 3D-printed uterus phantom) as a function of the connector position (i.e., “L”). Three samples were tested for each configuration. Mean curves and error bars (± standard deviation) are reported at selected volume values.

To characterise the mechanical behaviour of the device, 9 conditions were studied, combining three possible positions (i.e., “L” equal to 100, 75, and 50 mm) with different numbers of full rotations (1, 2, or 3). Results are reported in Fig. 4a, where data available in the literature for the ESM device^23^ are also mapped: the maximum average elongation and force at the point of failure of the connection are 15.1 N and 358 mm, respectively^23^.

The graphs reported in Fig. 4a can be used to extract the data summarised in Table 1. These data show that the maximum force values obtained are higher than the reference one (i.e., the ESM device^23^) for 3 full rotations at L = 100 and L = 75 mm (see also Fig. 4a top and middle). Therefore, this number of rotations was selected to finalise the design of the rotation mechanism.

**Table 1:** Evaluation of the connection strength through tensile testing: maximum force (mean value) for the 9 analysed configurations (three connector positions “L”, with 1 to 3 full rotations). Values higher than the reference from^23^ related to the ESM™ device are highlighted in bold. Data extracted from the graphs provided in Fig. 4a.

Connector position “L” [mm]	Number of full rotations	Maximum force [N]	Reference value [N]
100	1	4.05	15
	2	13.98	
	3	**25.95**	
75	1	5.24	
	2	13.51	
	3	**27.96**	
50	1	5.40	
	2	**15.32**	
	3	**15.32**	

The results of the tensile tests also demonstrate that the *connection strength* requirement is satisfied. This finding is particularly significant when considering the connector's working conditions, with the device placed inside the uterus during treatment. In this environment, the connector is subjected to various forces that may potentially compromise its integrity. However, our tests indicate that the connector possesses the necessary strength to withstand such forces, thereby ensuring its functionality and reliability in clinical applications.

However, to make the final decision concerning “L”, i.e., to select the most effective position of the connector on the rectal probe, the results from the ILP measurements were necessary. They are discussed in the following section.

### Intraluminal pressure results

Based on the mechanical test results, for the measurement of the ILP the number of connector full rotations was fixed to 3 and only the connector position design variable was evaluated (i.e., “L” equal to 100, 75, and 50 mm; Fig. 3c). The intraluminal pressure–volume graphs obtained are reported in Fig. 4b.

In unconfined (open air) conditions, independently of the connector position, after an initial increase, the pressure stabilises until the volume reaches 1500 ml (Fig. 4b, top). These open-air results are comparable to those available in the literature for the ESM device^23^, for which an average peak pressure of 12.4 N is reported at 300 ml, followed by a stable plateau at filling volumes up to 1200 ml.

In confined conditions and using the commercial simulator (Fig. 4b, middle), in all three “L” configurations, after an initial increase of up to 20 mmHg, the intraluminal pressure reaches a plateau when the volume of the balloon is at 150 ml. This is followed by another rise in pressure at 500 ml with different behaviours depending on “L”. When the connector is positioned at L = 100 mm, a steeper increase is observed, reaching a maximum pressure of 27.4 mmHg at the maximum filling volume (700 ml). The maximum pressures reached for L = 75 mm and L = 50 mm are 23 and 24.2 mmHg, respectively (Fig. 4b, middle). The 3D-printed uterus model results (Fig. 4b, bottom) are comparable with those of the commercial uterus model (Fig. 4b, middle) for low filling volumes; moreover, the 3D-printed model allows increasing the maximum testable volume up to 1500 ml compared to the commercial uterus. In all three “L” configurations, the pressure rises to peak values of around 18 mmHg (for filling volumes around 150 ml of water). Then, it stabilises on plateau values around 15.5 mmHg up to the final filling volume of 1,500 ml. Minor differences were detected between the three “L” configurations. It is necessary to mention that none of the specimens showed any leakage during the experiments. Therefore, the connector allows the probe cover to be inflated with up to 1,500 ml of fluid without a balloon rupture or the connection's weakening.

Concerning the confined conditions, it is worth explaining that a comparison with the data available in^23^ was not feasible due to the highly different testing conditions. Indeed, in^23^, three rigid aluminium uterus models with smaller internal cavities (100, 250, and 500 ml) were used, leading to ILP values around 400 mmHg. Therefore, these reference data are not reported in Fig. 4b, middle and top. Our device has demonstrated impressive integrity and functionality. Injecting 1500 ml of fluid in open air revealed its capability to handle huge volumes without compromising it.

For the ILP measured during the 4-h test in confined conditions (data are reported in Supplementary Fig. S6), a pressure drop of less than 2 mmHg is experienced during the first hour; then, the pressure inside the balloon remains constant for the following three hours. These results altogether confirm that the *hydraulic sealing* requirement was also achieved. Besides considering the requirements for the whole device, these ILP tests also assessed the in vitro* efficacy* of the BAMBI kit. Finally, concerning the “L” value, we selected an intermediate position for the connector (i.e., L = 75 mm). This choice allows the probe cover to be not too tight to the rectal probe when L = 50 mm, nor too loose when L = 100 mm. The shape of the inflated balloon also changes based on the “L” value: it tends to become closer to a sphere (when inflated in the open air) the more we increase “L”. Having the uterus not a spherical shape^34^, we adopted an intermediate value for “L”. Based on this final choice, the black signs shown in Fig. 1a were applied to the rectal probe.

### Further health technology assessment (HTA) evaluations

In the previous sections, the iterative design process and the mechanical characterisation of the innovative BAMBI connector were detailed, with results that demonstrated its equivalence with already approved similar devices. However, mechanical characterisations are not enough to demonstrate the fulfilment of all the requirements set. Additional analyses were performed.

**Figure 5:**
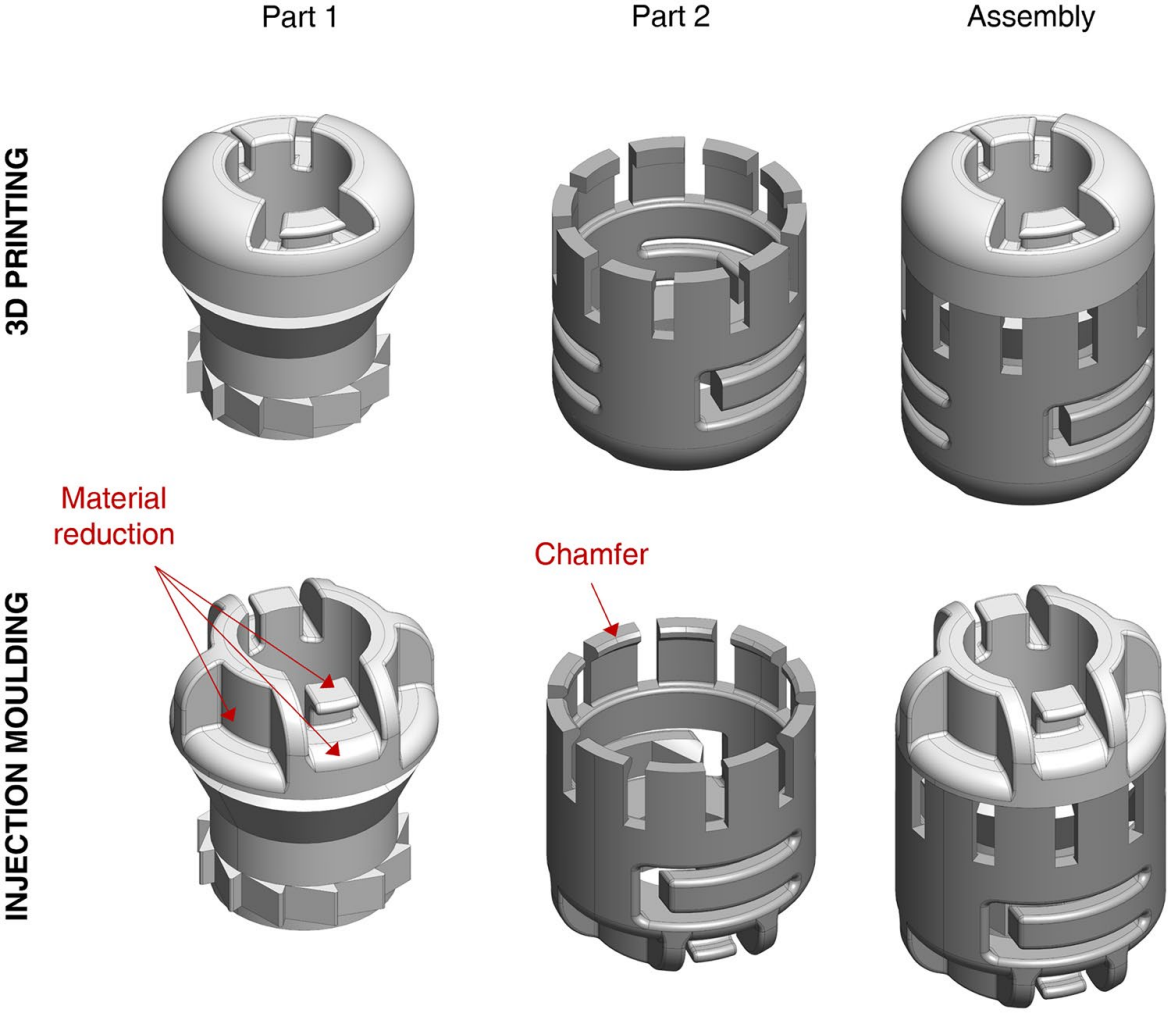
Possible redesigns of the two parts of the BAMBI connector for production with injection moulding (IM) and to reduce the amount of used material. The arrows indicate the main changes that consist of chamfers and cavities.

*Biocompatibility and sterilisation of the materials* are essential requirements for invasive medical devices and are related to the choice of material and manufacturing technology. The connector’s parts 1 and 2 could be manufactured using rigid or semi-rigid polymeric materials (plastics) already used for single-use medical devices in contact with human tissues, such as polyethylene (PE), polypropylene (PP), or polyoxymethylene (POM). They can also be easily sterilised with moist heat (i.e., autoclave) or ethylene oxide, typically used for temperature-sensitive materials. Besides, the developed concepts were 3D-printed in this study, but cheaper technologies can be used to manufacture the final product. A preliminary cost analysis (production cost) was performed on the connector, considering injection moulding (IM) as the selected technology. Before analysing costs, the two parts of the connector were redesigned to guarantee the possibility of producing them by IM and to reduce the amount of used material (Fig. 5). Although an optimal frugal approach would involve local production of the BAMBI device in the countries where it is more needed, a preliminary quotation to produce the connector by IM was requested to an Italian small and medium-sized enterprise (SME). The cost of a single connector (top and bottom) varies from €0.95 per unit for small batch production (1,000 units) to €0.33 per unit for larger quantities (30,000 units). These estimates were made considering an aluminium mould, but the unit cost can be further reduced by using a steel mould for higher production volumes. These estimates meet the 1€/piece requirement (*minimum cost*).

To contextualise production volumes, an initial estimate could be made by taking India as a reference country, which, according to the last trends^1^, is the second country (the first one is Nigeria) for the highest number of annual maternal deaths (24,000 in 2020). A recent study^35^ evaluated the cost-effectiveness of different UBT devices for guiding public health expenses and interventions. That study concluded that CBT solutions (such as the ESM) are economically sustainable and cost-saving compared to more expensive devices such as the Bakri. This study^35^ estimated that an annual cohort of almost 60,000 women would be eligible for UBT insertion after experiencing atonic PPH. This value was obtained starting from the annual number of deliveries in public health facilities (slightly more than 20,000,000). PPH incidence in India is around 3.6%, i.e., PPH affects 3.6% of all women giving birth in the country, which translates into almost 720,000 cases of deliveries. Among possible PPH causes, uterine atony is the most common. Following Indian guidelines, first-line intervention is based on uterotonic drugs and UBT devices are employed in unresponsive cases, estimated as 60,000 every year. A batch production of 30,000 units (€0.33 per unit) would theoretically be sufficient for almost 50% of the annual Indian need for UBT devices. These data also demonstrate the potential clinical implications of guaranteeing the spread and adoption of a low-cost and effective UBT kit such as BAMBI.

*Rapidity to assembly* and *ease of use* are fundamental requirements for any medical device, and even more so for emergency devices such as BAMBI, which aims to overcome the limitations of the improvised CBT devices currently used in LMICs. The fulfilment of these requirements has been evaluated in a simulated environment through usability tests conducted with medical and non-medical users. The details of these tests are not included here because they have already been described in a previous study by the same authors^22^. What is discussed here is how we have used the insights gathered from the tests discussed in^22^ to introduce further improvements in the connector design and, vice versa, how the in vitro tests described in this study helped elaborate the instructions for the training of the operators in the use of BAMBI.

**Figure 6:**
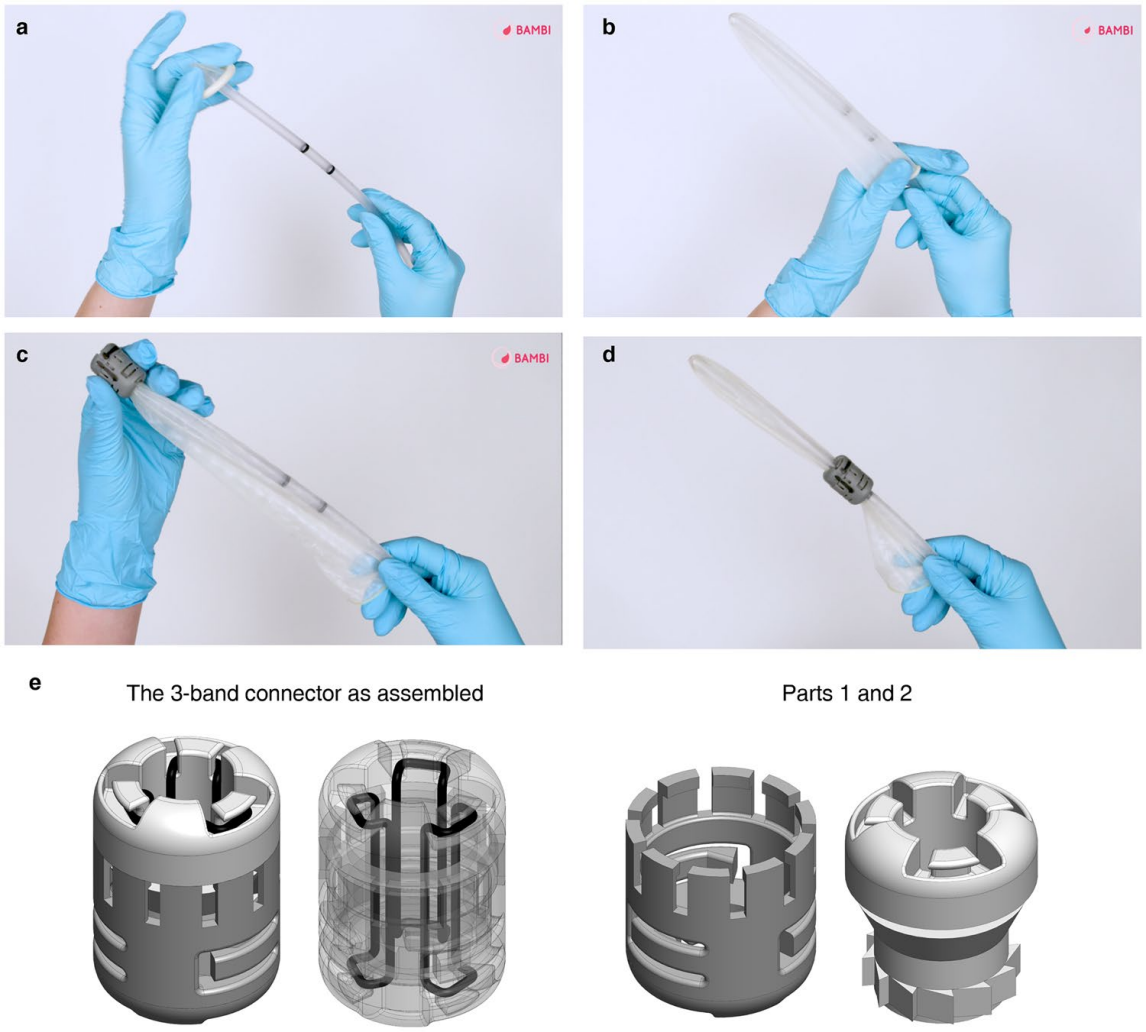
Frames from the video (see Supplementary Video S1) developed to train the potential end-users of the BAMBI kit and an improved version of the connector. The four frames correspond to the insertion step of the assembly procedure shown in Fig. 3b: (**a-b**) insertion of the probe cover on the rectal probe until the probe cover is fully unwrapped; (**c-d**) insertion of the connector from the top of the probe cover. The connector should slide until it reaches the position between the two black signs on the rectal probe. (**e**) Novel design of the connector with three elastic bands: as assembled (left) and its main parts (right). Increasing the elastic band number from two to three can reduce the number of full relative rotations of the two parts necessary to assemble the device and, consequently, the assembly time.

Indeed, for UBT devices, WHO guidelines suggest that appropriate training of health workers is required; at the same time, they recognise that the most effective approach to UBT training is not yet known and that this is an urgent priority knowledge gap to address^14^. Live session training is a more interactive modality, but it is only sometimes possible, especially in field hospitals in LMICs. Therefore, traditional paper IFU and video training are valid alternatives. Based on the experience gained by the authors during the in vitro tests (i.e., the mechanical characterisation and the ILP measurement), for example, the steps shown in Fig. 6a–d were conceived to be included in the video developed for the BAMBI training (see Supplementary Video S1) to explain how to insert the connector and fix the probe cover properly.

The intent to create a video was motivated by considering that today smartphone accessibility and availability and internet connection coverage are almost ubiquitous worldwide. Most of the global population owns mobile phones today. Based on an estimate, related to 2022, only less than 5% of the population has no mobile internet connectivity^36^. Such an increase in internet access has suggested health services via mobile phones or other mobile technologies (the so-called mHealth) as an essential means to reach peripheral and rural areas. Starting from these considerations, a recent study^36^ compared, for example, hands-on and video training as two alternative teaching methods for PPH management. No significant differences between the two modalities were found, suggesting that training by mobile media is a feasible and effective way to overcome the outreach gap in sub-Saharan Africa’s rural areas^36^. The BAMBI video training could be delivered via a mobile app integrated into the kit and accessible, for example, via a QR code on the outer packaging.

Together with the training modality, the usability tests presented by the authors in^22^ provided useful insights for improving the BAMBI design. Observation of users handling the device and analysis of use errors, combined with user comments, led to further thinking about a possible modification to the connector design. As the hydraulic sealing and the connection strength are achieved by wrapping the elastic bands around the probe cover, increasing their number from two to three could reduce the number of necessary complete rotations and, therefore, the assembly time without compromising the device’s performance. Based on these considerations, a new version of the connector was designed (Fig. 6e). Further testing will be required to assess the impact of this modification.

Together with the mechanical characterisation described in the previous section, these further evaluations confirm the potentiality of BAMBI as an effective and accessible UBT device to treat PPH. Given the social and economic determinants of the problem, a low-cost UBT device designed by adopting a frugal and user-centred approach, such as BAMBI, could indeed contribute to reducing inequalities in maternal health between countries, being a potentially *affordable* and *accessible* life-saving solution. Even if not specifically addressed in the present work, BAMBI also has the potential to be an *acceptable* device: UBT acceptability is already recognised by WHO guidelines, both for healthcare providers and women^14^, based on qualitative evidence provided by two systematic reviews^37,38^. More importantly, neither women nor providers preferred a particular type of UBT devices^14^. Providers in most of the studies used simple CBT devices, suggesting that cheap solutions are as acceptable as more expensive ones. However, further studies are necessary to extend the device validation and evaluate its effectiveness, safety, and reliability in real PPH emergencies.

## Conclusions

This paper presents BAMBI, a low-cost UBT kit developed for treating PPH, the worldwide main obstetric cause of maternal mortality. The kit is based on the CBT technique. However, unlike the improvised solution currently used in LRSs, it is equipped with an innovative connector to assemble the condom to the catheter more reliably and easily. The kit and, particularly, the connector, the main novelty of the kit, has been designed by adopting a frugal and user-centred approach. The mechanical characterisation tests have demonstrated that BAMBI is equivalent to approved similar devices, such as the ESM. Preliminary evaluations concerning the device production costs have shown that BAMBI also has the potential to be a cost-effective, affordable, and equitable solution for LRSs. Besides, further improved versions of the connector can be envisioned. Training aspects concerning the use of the device are also discussed, highlighting how the in vitro tests performed have helped elaborate the instructions for the video conceived for the training. Despite these results, further analyses, such as on-field clinical trials, are necessary to evaluate its effectiveness in real PPH emergencies.

### Supplementary Information

Supplementary Information 1.

Supplementary Video 1.

## Data Availability

The datasets generated during and/or analysed during the current study are available from the corresponding author upon reasonable request.
