# Supplementary material for: A novel low-cost uterine balloon tamponade kit to tackle maternal mortality in low-resource settings
Source: Sci Rep. 2024 May 1;14:10004. doi: 10.1038/s41598-024-60064-z (PMC11063067; doi:10.1038/s41598-024-60064-z)
Supplement: Supplementary file 1 — Supplementary Information 1. [file 41598_2024_60064_MOESM1_ESM.pdf]

A novel low-cost uterine balloon tamponade kit to tackle maternal mortality in low-resource settings

Sara Candidori[1], Kasra Osouli[2], Serena Graziosi[1*], Alberto Antonio Zanini[3], Maria Laura Costantino[2], Francesco De Gaetano[2]

1 Department of Mechanical Engineering, Politecnico di Milano, Via La Masa 1, Milan 20156 Italy
2 Department of Chemistry, Materials and Chemical Engineering "G. Natta", Politecnico di Milano, Piazza Leonardo da Vinci 31, 20133 Milan, Italy
3 Obstetrician Gynaecologist, Freelance Professional, Milan, Italy

*Corresponding author email: serena.graziosi@polimi.it

Supplementary Figure S1

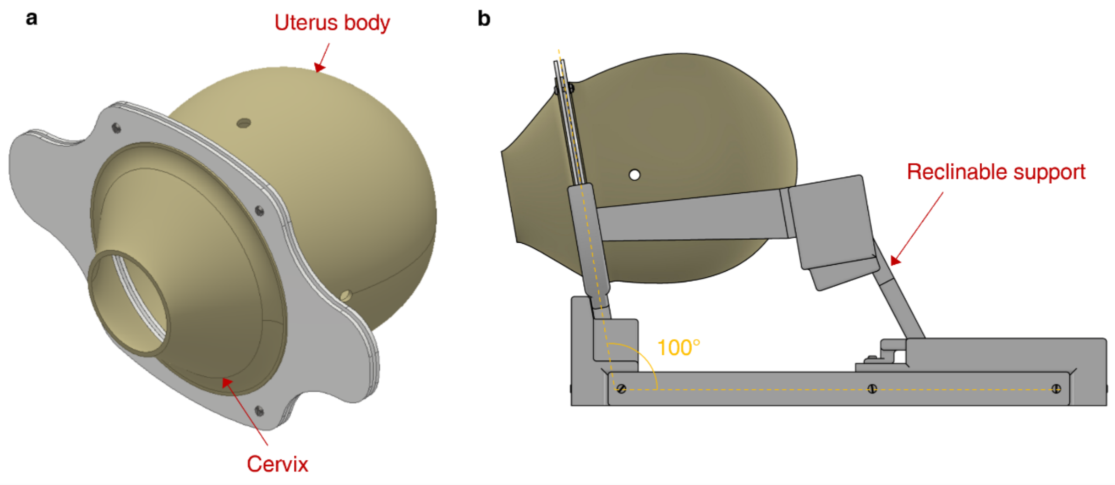

Figure S1. The digital model of the 3D-printed uterus phantom. (**a**) The two main parts are the uterus body and the medium cervix. (**b**) The uterus phantom as it is mounted on the reclinable support. This support has been conceived to test different organ orientations: it mimics three positions of the mother's body after the delivery. This image shows the orientation at 100° because it is used to perform the intraluminal pressure tests. Further details about the 3D-printed uterus phantom are available in [s1].

Supplementary Note - Preliminary concepts

This supplementary note details the initial phase of the design process. In this phase, multiple solutions were developed for a preliminary validation activity. Functional prototypes were manufactured for each solution, and preliminary tests were performed to assess the feasibility of each solution and select the most promising one for a detailed analysis.

Components and assembly procedures of the proposed connectors

In the initial phase, three solutions were developed. They are (**Fig. S2**) the "interlocking connector", the "hinge connector", and the "rubber band connector". CAD models were created for each solution,

and prototypes were manufactured using 3D printing technologies. The procedures for their assembly, use, and testing were also defined.

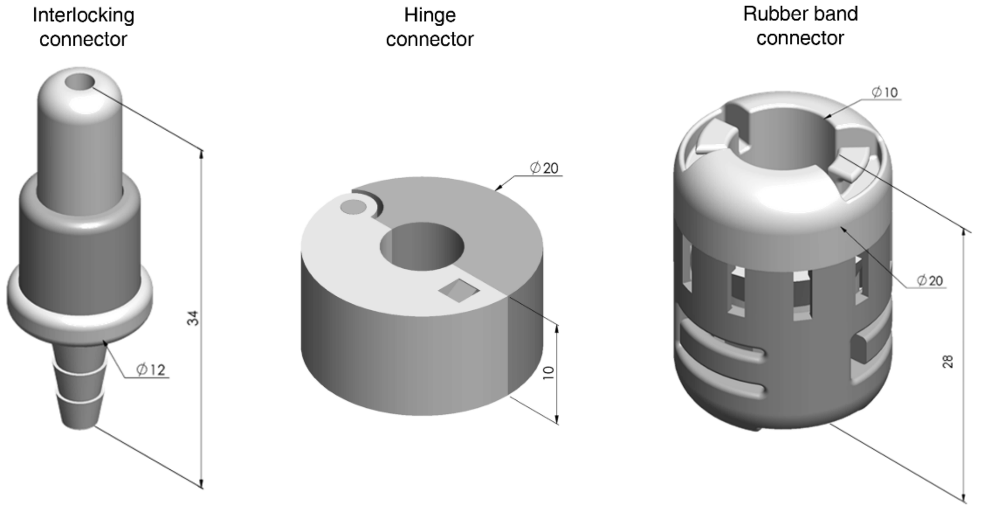

Figure S2. Three initial concepts for the connector element of the BAMBI device: 1) interlocking, 2) hinge, 3) rubber band. Dimensions are expressed in millimetres.

The main manuscript provides all the details about the rubber band connector because it is the selected solution. Hence, in the supplementary material, we focus on the other two: the "interlocking connector" and the "hinge connector",

Interlocking connector

The interlocking connector comprises a hollow lower body (part 1) and a pierced upper head (part 2), shown in **Fig. S3a**. The proximal end of part 1 is threatened to allow the attachment of the catheter/probe cover. The enlargement in the medial position enables the housing of the head at the end of the assembly. The assembly procedure is shown in **Fig. S3b**: the unrolled probe cover is inserted from the distal end of part 1, and that assembly is passed through the perforated head (part 2) fixed to part 1 through two pins that ensure sealing. Specifically, part 2 is rotated so that the pins run along two guides and fit into a cavity located at the guide ending, ensuring the fixing.

Hinge connector

The hinge connector consists of two hollow semi-cylindrical bodies, parts 1 and 2, shown in **Fig. S3c**, coupled by a hinge connection that allows the relative rotation of the two parts around a fixed axis of rotation. The assembly of the parts is based on a standard snap-fit solution: at one extremity of part 2, there is a protrusion with fins that flex when inserted into the slot of part 1. Once fully inserted, the fins deflect outwards back to their neutral position and the connection is achieved. This solution is easy to use: it is sufficient to unroll the probe cover over the rectal probe, position the connector over the probe cover and apply a slight pressure to close the clip (**Fig. S3d**).

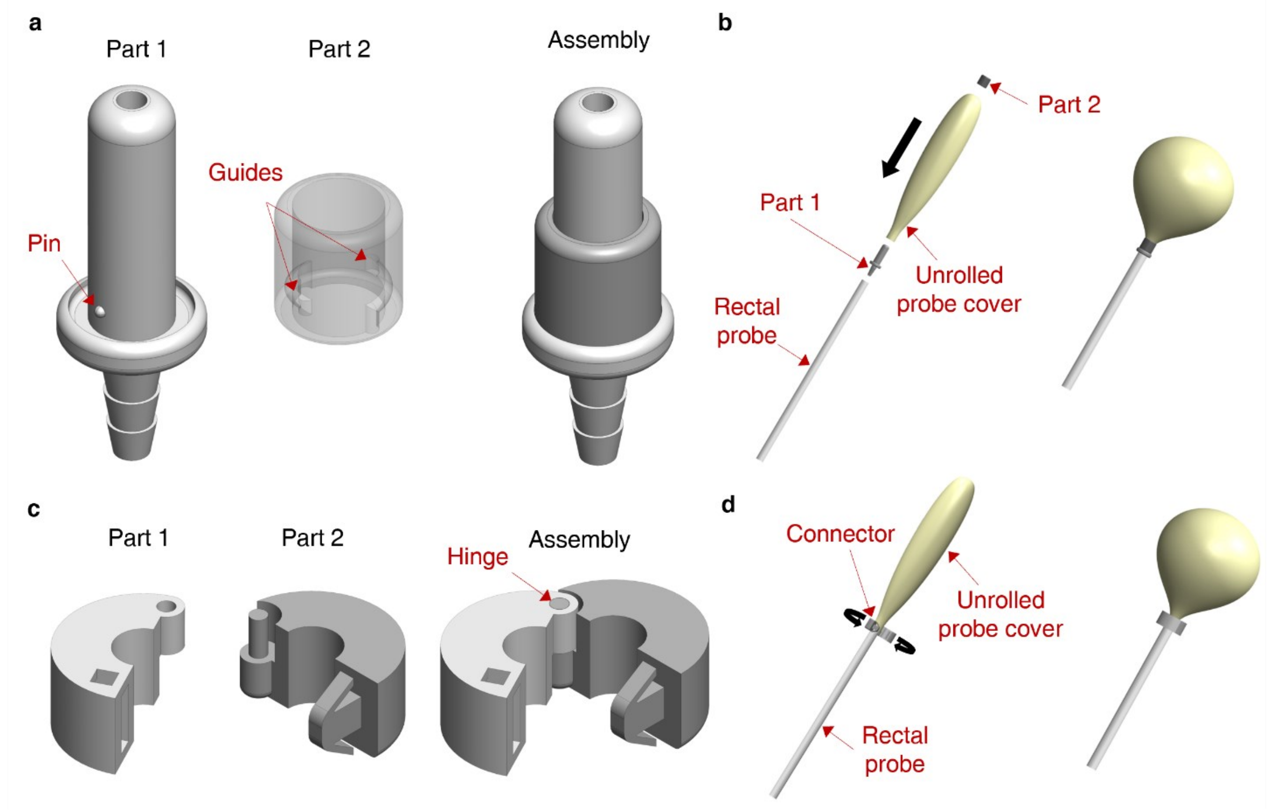

Figure S3. Interlocking and hinge connectors. (**a**) Components of the interlocking connectors: parts 1-2 and assembly. (**b**) Assembly procedure of the interlocking connector. (**c**) Components of the hinge connector: parts 1-2 and assembly. (**d**) Assembly procedure of the hinge connector.

Preliminary testing of the initial concepts – methods

During this initial phase, three preliminary tests were planned and performed to assess the feasibility of each designed solution (**Fig. S.1**). These tests included a leak test to verify that the device performs as expected and does not leak during regular use, a simulated use test to confirm that the device can be assembled and used reliably and a tensile test to evaluate the connection strength.

The leak test started with the assembly of the device. Then, the balloon was filled with water up to 800 ml in 50 ml steps using a 500 ml syringe, and eventual fluid leakages were visually detected and annotated but not quantified. The experimental setup was the same as the intraluminal pressure measurement in open air (see **Fig. 2b**), even if the pressure was not detected in these preliminary tests.

The simulated use test, performed by the research team, consists of 1) the assembly of the device, 2) its insertion and inflation inside the commercial simulator Prompt Flex PPH Module LIM-80101 (Limbs and Things Inc.^{TM}), and 3) its deflation and extraction from the simulator. At the beginning of the test, all the kit components were positioned on a table, together with the uterus simulator. Then, the device assembly started, based on the procedure shown in **Fig. 3b** for the rubber band connector and **Fig. S.3d** for the hinge connector. The balloon was inserted inside the uterus simulator and inflated up to 800 ml in 50 ml steps using a 500 ml syringe. Then, it was deflated and extracted from the simulator. The integrity of the device was monitored visually during the entire procedure. The experimental setup was similar to that described for the intraluminal pressure measurement in confined conditions (see **Fig. 2c**). The probe cover breaking point (bursting volume) was evaluated

using the rubber band elastic connector. The assembled device was filled with water until the probe cover burst. The bursting volume was annotated. All tests were repeated three times.

Preliminary tensile tests were performed to compare the two concepts that passed the previous tests (hinge and rubber band connector) and to verify if the *connection strength* requirement was satisfied. The setup used is the same as described in the manuscript (see the **Methods** section).

All tests were repeated three times.

Preliminary testing of the initial concepts - Results

The hinge and rubber band connectors did not show fluid leakages during balloon inflation for filling volumes up to those used in clinical practice (800 ml). They can also be easily assembled and used without detected difficulties. The interlocking design did not pass the preliminary tests since fluid leakages were observed at low filling volumes (100 ml), becoming copious for higher volumes; moreover, this solution was deemed challenging to assemble. Some pictures taken during the leak tests are shown in **Fig. S4.** Before the second testing phase, the probe cover breaking point (burning volume) was 10,000 ml ± 730 ml, far above the reference value of 5,000 ml of the ESM devices[s2].

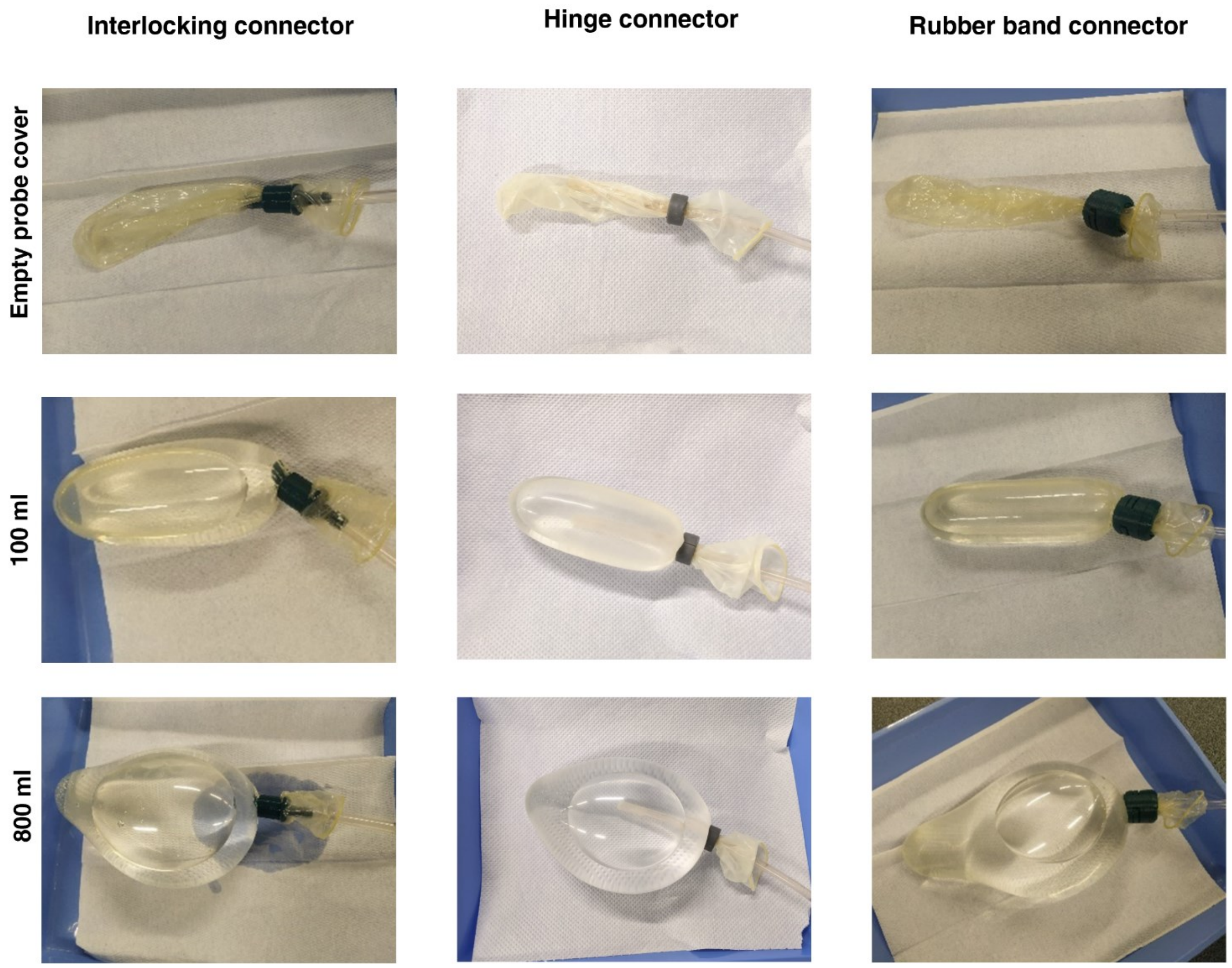

Figure S4. Leak test on the three initial concepts: while the hinge and rubber bands connectors did not show any fluid leakage for filling volumes up to 800 ml, leakage was observed for the interlocking connector.

The two prototypes deemed compliant with initial tests moved to the second phase of preliminary checks: the purpose was to provide an initial evaluation of the mechanical properties of the devices.

The force-displacement curves of the two solutions are shown in **Fig. S5**. The elongation and strength at the time of connection failure were 398.32 ± 54.26 mm and 10.07 ± 2.15 N for the rubber band connector, while values of 150.59 ± 14.17 mm and 3.59 ± 0.47 N were recorded for the hinge connector. Therefore, the rubber band connector could withstand higher forces than the hinge-based design, which was, therefore, discarded.

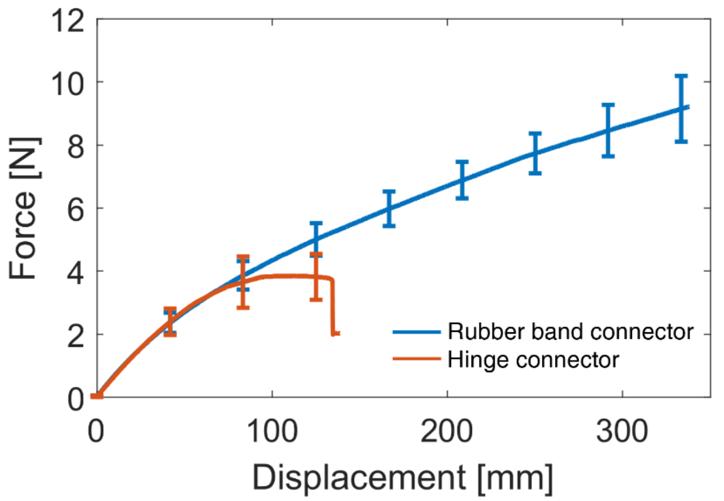

Figure S5. Force-displacement curves of the hinge- and rubber band-based assembled devices. Three samples were tested for each configuration. Mean curves and error bars (± standard deviation) are reported at selected volume values.

It should be noted that the maximum tensile force value of the rubber band connector was below the target value at this design stage. Before moving to the detailed analysis of the chosen solution, we tried to increase its connection strength by analysing the rubber band elements. Commercial hair comb elastics with a diameter of 15 mm were initially used in the connector concept. Once this design had been selected, they were replaced by dental brace elastics with a diameter of 9.5 mm. Contrary to hair comb elastics, orthodontic bands are medical devices: they are already approved to be used inside the human body and are sold sterile.

Supplementary Figure S6

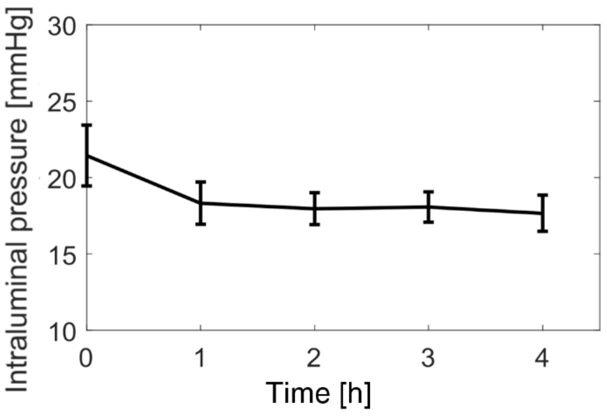

Figure S6. Variation of the intraluminal pressure (ILP) as a function of time. The BAMBI device was inflated in confined conditions using the commercial uterus model (**Fig. 2c**) up to the maximum volume of 700 ml and left inside the model for 4 h. The ILP remains almost constant during the test. The mean curve and error bars (± standard deviation) are reported.

Supplementary references

s1. Candidori, S. et al. Design and 3D printing of a modular phantom of a uterus for medical device validation. *Rapid Prototyp. J.* **29**, 7–20 (2023).

s2. Mollazadeh-Moghaddam, K. et al. Mechanical Properties of the Every Second Matters for Mothers-Uterine Balloon Tamponade (ESM-UBT) Device: In Vitro Tests. *Am. J. Perinatol. Reports* **09**, e376–e383 (2019).
